# Influence of Pilocarpine Eyedrops on the Ocular Biometric Parameters and Intraocular Lens Power Calculation

**DOI:** 10.1155/2023/7680659

**Published:** 2023-07-07

**Authors:** Ruxin Gao, Jinkun Liu, Xiaojie Zhou, Luping Huang, Weiyi Huang, Yingying Xue, Fei Wang, Songjian Gong, Renyi Wu, Yuhong Wang

**Affiliations:** ^1^Department of Ophthalmology, Eye Institute & Affiliated Xiamen Eye Center, School of Medicine, Xiamen University, Xiamen 361001, China; ^2^Department of Ophthalmology, Fujian Provincial Key Laboratory of Corneal & Ocular Surface Diseases, Xiamen 361001, China

## Abstract

**Objective:**

To evaluate the influence of pilocarpine eyedrops on the ocular biometric parameters and whether these parameter changes affect the intraocular lens (IOL) power calculation in patients with primary angle-closure glaucoma (PACG).

**Methods:**

Twenty-two PACG patients and fifteen normal subjects were enrolled. Ocular biometric parameters including the axial length (AL), anterior chamber depth (ACD), lens thickness (LT), mean keratometry (Km), and white-to-white distance (WTW) were measured by using a Lenstar LS 900 device before and at least 30 minutes after instillation of 2% pilocarpine eyedrops. Lens position (LP) was calculated, and the IOL power prediction based on the ocular biometric parameters was performed using the Barrett Universal II, Haigis, Hoffer Q, Holladay I, or SRK/T formulas before and after pilocarpine application.

**Results:**

In both PACG and normal groups, pilocarpine eyedrops induced a slight but statistically significant increase in the mean AL (0.01 mm for both groups) and mean LT (0.02 mm and 0.03 mm, respectively) but a significant decrease in the mean ACD (0.03 mm and 0.05 mm, respectively) and mean LP (0.02 mm and 0.04 mm, respectively). No significant changes in the mean Km and WTW were noticed in both groups. In addition, the IOL power calculation revealed insignificant changes before and after the pilocarpine instillation in both groups, regardless of the formula used.

**Conclusions:**

Pilocarpine eyedrops can induce slight changes in the ocular biometric parameters including the AL, ACD, LT, and LP. However, these parameter changes will not result in a significant difference in IOL power estimation.

## 1. Introduction

Lens extraction, combined with the intraocular lens (IOL) implantation, has been the standard procedure for cataract treatment for decades. With the development of both surgical techniques and IOL design, modern cataract surgery provides a much improved visual outcome to meet patients' increasing expectations of visual functional restoration. In addition, it is now generally acknowledged that the morphological change of the crystalline lens as well as the change of its position inside the eyes plays an important role in the pathogenesis and progression of primary angle-closure glaucoma (PACG) [1]. Lens removal, regardless of any cataract formation, has been proven to be effective in widening the angle of the anterior chamber, and hence, reducing the risk of angle closure and the intraocular pressure (IOP) rise [2]. Clear lens extraction is even recommended as a treatment option under certain scenarios of PACG onset [3, 4]. To achieve an ideal postoperative visual outcome following cataract surgery, it is essential to calculate the power of the IOL to be implanted precisely before each surgery to minimize the postoperative refractive error. The accuracy of IOL power calculation is highly dependent on the biometric measurements of the eyeball [5, 6].

A series of instruments with different accuracy profiles are currently available in the clinical practice to measure the ocular biometric parameters. Lenstar LS 900 (Haag-Streit AG, Koeniz, Switzerland) is an optical biometry device to measure these parameters based on the technique of optical low coherence reflectometry (OLCR) with an 820-nm superluminescent diode [7, 8]. It is able to obtain the values of axial length (AL), anterior chamber depth (ACD), lens thickness (LT), mean keratometry (Km), central corneal thickness (CCT), white-to-white (WTW) distance, and pupil diameter (PD) in a single shot within a short time with good repeatability and reproducibility [9–11]. The IOL power estimation based on these ocular biometric parameters can be instantly conducted using an integrated calculator with multivariable IOL power prediction formulas.

PACG is still one of the major glaucoma subtypes worldwide, and the current estimated population with PACG is 17.14 million, with 12.30 million in the Asia area [12]. The number of PACG suspects that have occludable anterior chamber angles is even much bigger. It is further reported that nearly half of all PACG patients are of Chinese descent [13]. In Asian countries, it is a routine management for the PACG patients and suspects to be treated with pilocarpine eyedrops on a frequent basis to treat or prevent angle closure. Pilocarpine ophthalmic solution has been used for the treatment of PACG since the 1970s, primarily based on its miotic effect.

A recent clinical application of this agent is to manage presbyopia in the elderly [14]. Accommodation alteration of the eye, as one of the pharmacological effects of pilocarpine, is often seen in patients under treatment, which involve the axially thickening of the lens [15], the increase of the global AL [16], the forward movement of the lens [17], and the shallowing of the anterior chamber depth [18]. These changes may potentially influence the ocular biometric measurement and may therefore result in an alteration in the IOL power calculation based on these parameters. So far, studies on the influence of pilocarpine on the ocular biometric measurements and IOL power estimation are still rare, especially on those PACG patients who are the potential users of pilocarpine. In the present study, we investigated the influence of pilocarpine eyedrops on the changes in ocular biometric parameters measured by the Lenstar LS 900 and the changes in IOL power calculation carried out by using five formulas which are routinely used in the clinic including the Barrett Universal II, Haigis, Hoffer Q, Holladay I, and SRK/T formulas.

## 2. Materials and Methods

### 2.1. Study Design and Participants

This observational study comprised PACG patients who were 40 years old or above and were scheduled for filtration surgeries from October 2021 to March 2022 in a local eye center. Age-matched, healthy subjects without any history of major ocular diseases were also enrolled in the study as control. The study adhered to the tenets of the Declaration of Helsinki throughout the entire data collection process, and the ethical committees of both eye clinics approved the study. Consent to use their medical data for this research was given by all participants.

The clinical data were obtained from all patients through a comprehensive ophthalmologic examination, at least including objective (KR-800, TOPCON, Guangdong, China) and subjective refraction (NIDEK/AOS1500, Wuhan, China), assessment of the anterior chamber angle by gonioscopy, IOP measurement (Canon TX-20, Kawasaki, Japan), fundus analysis based on the photos taken by a digital retinal camera (KOWA, nonmyd WX, Tokyo, Japan), and optical coherence tomography (OCT) (OCT-5000, Carl Zeiss, Dublin, California, USA). The visual field defect of the suffering eye was measured with a Humphrey Field Analyzer (860, Carl Zeiss, Dublin, CA, USA). PACG was diagnosed based on closure of the anterior chamber angles with glaucomatous optic neuropathy and corresponding visual field loss.

Every participant underwent a gonioscopy examination by an experienced glaucoma specialist (Y Wang). Only the PACG eyes with functional trabecular meshwork visible in 2 quadrants or less were enrolled in our study. The exclusion criteria were as follows: (1) history of any ocular surgery; history of laser peripheral iridectomy or iridoplasty; (3) known ocular abnormality that could affect biometry measurement including corneal edema, corneal pannus, macular edema, dense posterior subcapsular cataract, and so on; (4) wore contact lens recently; (5) allergy to pilocarpine; (6) dropped out voluntarily because of severe drug side effects or failure to perform the measurements at the specific time; (7) inability to perform the biometry measurements accurately; and (8) iris anomaly, especially after an acute attack of PACG. In patients with PACG in both eyes, only the eyes with lower severity were chosen. The right eyes of normal subjects were selected as the control group.

### 2.2. Data Acquisition

For each subject, ocular biometric parameters including AL, ACD, LT, Km, CCT, WTW, and PD were obtained using the Lenstar LS 900 device by the same experienced examiner under identical light conditions. Repeat measurements were conducted for at least 3 times for each eye, and the mean value of each parameter was recorded. The ACD was defined as the distance between the cornea endothelium and the anterior surface of the lens. The lens position (LP) was defined and calculated as the values of ACD + 1/2LT [19]. The IOL power for a one-piece PCB00 IOL with an A constant of 118.8 (Johnson and Johnson Vision, Florida, USA) was calculated by using the Lenstar LS 900 using the Barrett Universal II, Haigis, Hoffer Q, Holladay I, and SRK/T formulas.

Pupillary constriction was achieved with the topical application of 2% pilocarpine nitrate eyedrops (Freda Pharmaceutical, Shandong, China) at 15 min intervals. After 30–45 minutes, ocular biometric measurements were taken because the effect of pilocarpine on the ciliary muscle works maximally in 30 minutes to 60 minutes without IOP fluctuation [20–22].

### 2.3. Statistical Analysis

Continuous variables are represented by the mean ± standard deviation [23]. The statistical software of SPSS (SPSS Inc., Chicago, USA) was used for data analysis. A minimum sample of 13 eyes was estimated to be necessary to detect a mean ACD difference of 0.20 mm between premiosis and postmiosis measurements with a 0.05 *α* error and a 0.10 *β* error, assuming 0.25 mm SD in each group.

Student's *t*-test was used for comparing the normally distributed paired data of ocular biometric measurements and IOL powers. Otherwise, Wilcoxon signed-rank tests were used for the comparison. The correlation between variables was determined by the Pearson or Spearman correlation test according to the normality of the data distribution. The Fisher's exact test was used to compare the coincidence of IOL power calculated by the five formulas, respectively, and the coincidence of IOL power between PACG and the control subjects. A deviation in the absolute value up to 0.25 D in the IOL power calculation was considered unacceptable. The level of significance was set at *p* < 0.05.

## 3. Results

### 3.1. Study Population

A total of 22 eyes from 22 PACG patients (mean age 63.42 years, range from 51 to 76 years) and 15 eyes from 15 ocularly healthy patients (mean age 59.10 years, range from 44 to 70 years). The baseline demographic and clinical characteristics are summarized in Table 1. When compared with those of normal subjects, eyes of PACG patients had significantly worse mean visual acuity (*p* < 0.001), smaller ACD (*p* < 0.001), LP (*p*=0.001), and pupil diameter (*p*=0.002) but greater LT (*p*=0.002).

### 3.2. Influence on the Ocular Biometric Measurements

**Figure 1:**
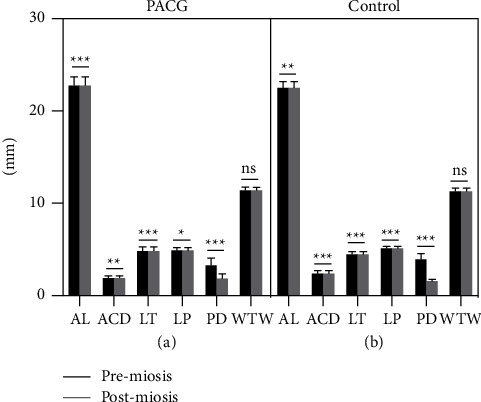
Influence on the ocular biometric parameters in PACG and normal subjects. The error bars represent the standard deviation of measurements for 22 PACG patients (a) and 15 normal subjects (b).

Changes in the ocular biometric parameters before and after pilocarpine eyedrops instillation are summarized in Table 2 and Figure 1. In both PACG patients and normal subjects, application of pilocarpine eyedrops induced a slight but statistically significant increase in the mean AL (0.01 mm for both groups) and mean LT (0.02 mm and 0.03 mm, respectively), but a statistically significant decrease in the mean ACD (0.03 mm and 0.05 mm, respectively), mean LP (0.02 mm and 0.04 mm, respectively), and mean PD (1.47 mm and 2.32 mm, respectively). No significant changes in the mean Km and WTW were noticed both in PACG and normal subjects. A borderline significant (*p*=0.049) increase in CCT was only seen in the PACG group. In addition, neither the absolute value nor the percentage (data not shown) of the changes in ACD, LT, and LP in PACG patients was statistically different from that in the normal participants (all *p* > 0.05), despite the fact that a significant difference of those parameters existed between the two groups at baseline. In both groups, none of the changes in the AL, ACD, LT, and LP was associated with their corresponding values at baseline (all *p* > 0.05, Spearman's test).

### 3.3. Influence on the IOL Power Calculation

**Figure 2:**
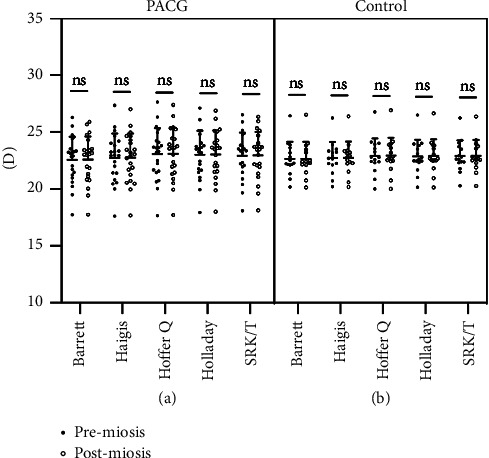
Influence on IOL power calculated using five formulas in PACG patients and normal subjects. The error bars represent the standard deviation of measurements for 22 PACG patients (a) and 15 normal subjects (b).

Results of the IOL power calculation by the Barrett Universal II, Haigis, Hoffer Q, Holladay I, or SRK/T formulas are shown in Table 3 and Figure 2. In both groups, changes in the IOL power before and after the pilocarpine eyedrops instillation were all statistically insignificant (all *p*  > 0.05) regardless of the formulas used for calculation. In addition, neither the absolute value nor the percentage (data not shown) of the changes in the IOL powers were insignificant in both groups, regardless of the formula used.

The percentage of eyes which had a deviation in the IOL power calculation of not more than 0.25 D (absolute value) after pilocarpine eyedrops instillation is summarized in Table 4. There was also no statistical difference (all *p* > 0.05) in the coincidence of IOL power calculated by the five formulas both in the PACG and normal participants, although the Barrett Universal II formula and the SRK/T formula had similar relatively high coincidence rates in both groups.

## 4. Discussion

To the best of our knowledge, this is the first study to investigate the influence of pilocarpine eyedrops on the measurement of ocular biometric parameters and IOL power calculation in patients with PACG and normal subjects. Our study showed that although there were slight changes in some of the parameters, the IOL power recommended for a cataract surgery did not differ significantly after pilocarpine application.

Pilocarpine is now widely used in the treatment of glaucoma, especially for PACG patients. In addition, recent approval had been issued by the Food and Drug Administration (FDA) for its use in the presbyopia improvement among the elderly [14]. It is well known that pilocarpine induces ocular accommodation which may alter the measurement of ocular biometry and refractive statue. AL has a substantial impact upon the IOL power calculation according to the algorisms for calculation; nevertheless, the magnitude of the changes in AL was not big enough to cause a significant shift in the IOL powers estimation by the five formulas used in our study. Moreover, we did not find any correlation between the changes in AL and the changes in IOL power calculation, either.

A recent research showed a mean elongation in AL of 0.03 mm after pilocarpine eyedrops use in healthy adults (mean age of 32.97 ± 5.21 years) when measured with an optical biometer AL-Scan [24]. Shao et al. [25], also found a mean increase in the AL of 0.04 mm in pseudophakic eyes (age range 49 to 84 years) induced by pilocarpine application. One possible explanation to this elongation of AL during the accommodation, as Drexler et al. [26] proposed, is that the contraction of the ciliary muscle pulls the choroid and sclera adjacent to the ciliary muscle forward and inward at the same time, resulting in a decreased global circumference at the equator, forcing the rearward movement of the posterior portion of the globe to maintain a constant ocular volume. Furthermore, one may speculate that the eyedrops may cause an increase in the corneal thickness due to the transient epithelial edema induced by the eyedrops itself, a phenomenon that has been reported elsewhere when some miotic eyedrops was instilled to the eyes [27]. However, this cannot be the case in the present study, as shown in Table 2 that the CCT change contributed negligibly to the increase of the AL in the PACG patients, and in the normal subjects, the CCT value even decreased after the use of pilocarpine.

It has been shown in some research that the pilocarpine-induced accommodation would reduce the depth of the anterior chamber but increase the thickness of the len. Grzybowski et al. [28, 29], confirmed that a small increase in central LT occur and was associated with a large increase in accommodative amplitude during accommodation on the basis of stringent image registration criteria, as predicted by the famous Schachar mechanism of accommodation, which states that there is an increase in equatorial zonular tension associated with relaxation of the anterior and posterior zonules, and the increase in zonular tension causes an increase in equatorial lens diameter, peripheral lens surface flattening with a resulting negative shift in spherical aberration, and counterintuitively, an increase in central lens thickness while the whole lens remains stable. To be specific, Abramson et al. [30], found a profound ACD decrease (0.24 mm) and an LT increase (0.18 mm) using A ultrasound in 5 young healthy participants (age range 21 to 26 years old) with preserved accommodation. Slight smaller changes in ACD (−0.26 mm) and LT (+0.24 mm) measured by an ultrasound were observed in a group of glaucoma patients with a mean age of 40 years [21]. When measured by Pentacam, the decrease in the mean ACD was only 0.08 mm in healthy phakic young adults after pilocarpine eyedrop instillation [24]. When partial coherence interferometry was used, a slight increase in LT (0.05 ± 0.46 mm) was noticed after pilocarpine eyedrop instillation in a group of emmetropic presbyopic subjects (age range 51 to 62 years) [31]. The difference in the LT change may at least partially reflect the ability of accommodative ciliary muscle contraction which in theory declines with age [32, 33]. Such a change in lens morphology and positioning are known as the risk factor for the pathogenesis of some specific glaucoma subtype, e.g., malignant glaucoma, another name for ciliary block glaucoma [34, 35].

About 2 mm pupil diameter effect has been demonstrated in human using a 2% ophthalmic pilocarpine solution for longer than an hour [22]. In our study, ophthalmic pilocarpine administration induced a significant decrease in pupil diameter both in PACG patients and normal subjects. The mean baseline pupil size in the PACG group was statistically significantly smaller than in the normal group, which is consistent with at least two other studies [36, 37]. The amplitude of pupil contraction values was statistically significantly higher in control subjects when compared to the patients with PACG (*p* < 0.001) in our study. The exact mechanisms responsible for the pupillary changes in glaucoma are not yet completely understood. The change of pupil dynamics and the rigidity and fibrosis caused by iris sphincter muscle involvement and relative iris autonomic dysfunction in the eye of glaucoma maybe the reasons [37, 38].

Formulas for IOL power prediction before cataract surgery are primarily based on the ocular biometric parameters. For example, the 3^rd^ generation IOL power calculation formulas (Hoffer Q, Holladay I, and SRK/T) use only corneal curvature radius and AL to calculate the IOL power [39, 40], while the 4^th^ generation formulas (Haigis and Barrett Universal II) improve the calculation methods by incorporating more parameters including ACD, LT, and WTW. The accuracy of those formulas under different clinical settings is different [41–46]. The influence of mydriatics on IOL power calculation has been reported elsewhere [47, 48]. In the present study, we observed that the overall mean change in the IOL power calculation after the use of pilocarpine eyedrops was rather insignificant both in PACG patients and normal subjects, with a relatively small portion of revealed an IOL power changes exceeding 0.25 D (see Table 4) which may have potential clinical relevance. Among the formulas used in the study, Barrett Universal II and SRK/T had a relatively high coincidence rate of IOL power before and after pilocarpine use. Further studies with an increased number of participants are needed before any recommendation could be made for the patients who had used pilocarpine eyedrops prior to a cataract surgery.

It has to be admitted that this study had several limitations. First, the number of the subjects we recruited in a single eye center was relatively small. Furthermore, since all participants received only several doses of pilocarpine before the second Lenstar measurement was taken, such a result might not be able to reflect the long-term effect of pilocarpine on the ocular biometric measurement alteration, which may very possibly occur on patients who use this eyedrops frequently. Research on the long-term effect of pilocarpine on the ocular biometric change is of clinical interest because of the potentially wide use of this agent in the treatment of presbyopia.

## 5. Conclusions

In summary, our study suggested that pilocarpine eyedrops may induce slight changes in the ocular biometric parameters including AL, ACD, LT, and LP shortly after drug application. However, these changes will not result in a significant difference in IOL power calculated using the Barrett Universal II, Haigis, SRK/T, Hoffer Q, or Holladay I formulas.

## Figures and Tables

**Table 1:** Baseline demographic and clinical characteristics.

Characteristics	PACG	Normal subjects	*p* values
Age (years)	63.42 ± 7.5	59.10 ± 6.5	0.092
Male, *n* (%)	10 (45.5%)	5 (33.3%)	0.514
UDVA	0.51 ± 0.24	0.85 ± 0.17	<0.001
SE (D)	0.05 ± 1.90	−0.87 ± 0.97	0.363
IOP (mmHg)	14.78 ± 3.78	15.67 ± 2.45	0.436
AL (mm)	22.94 ± 0.90	22.67 ± 0.66	0.415
ACD (mm)	1.95 ± 0.25	2.43 ± 0.31	<0.001
CCT (mm)	545.25 ± 24.80	535.53 ± 28.76	0.260
LT (mm)	4.94 ± 0.36	4.54 ± 0.29	0.002
LP (mm)	4.96 ± 0.27	5.24 ± 0.22	0.001
PD (mm)	3.45 ± 0.76	4.02 ± 0.53	0.018

ACD, anterior chamber depth; AL, axial length; CCT, central corneal thickness; D, diopter; IOP, intraocular pressure; LP, lens position; LT, lens thickness; PACG, primary angle-closure glaucoma; PD, pupil diameter; SE, spherical equivalent; UDVA, uncorrected distance visual acuity.

**Table 2:** Influence of pilocarpine eyedrops on the ocular biometric parameters in PACG and normal subjects.

Parameters	Group	Baseline	After eyedrops	Mean difference ± SD	95% CI for mean difference	*p* values
AL (mm)	PACG	22.94 ± 0.90	22.95 ± 0.90	0.01 ± 0.02	0.01, 0.02	0.001
	Control	22.67 ± 0.66	22.68 ± 0.66	0.01 ± 0.01	0.00, 0.01	0.010

CCT (mm)	PACG	545.25 ± 24.80	546.75 ± 23.65	1.90 ± 4.60	0.01, 3.72	0.049
	Control	535.53 ± 28.76	534.80 ± 28.93	−0.73 ± 2.89	−2.33, 0.87	0.342

ACD (mm)	PACG	1.95 ± 0.25	1.92 ± 0.26	−0.03 ± 0.05	−0.05, −0.01	0.003
	Control	2.43 ± 0.31	2.39 ± 0.30	−0.05 ± 0.04	−0.07, −0.02	<0.001

LT (mm)	PACG	4.94 ± 0.40	4.95 ± 0.40	0.02 ± 0.02	0.01, 0.02	<0.001
	Control	4.54 ± 0.29	4.56 ± 0.29	0.03 ± 0.02	0.02, 0.04	<0.001

LP (mm)	PACG	4.96 ± 0.27	4.94 ± 0.28	−0.02 ± 0.04	−0.04, 0.00	0.027
	Control	5.24 ± 0.22	5.21 ± 0.21	−0.04 ± 0.03	−0.05, −0.02	0.001

Km (D)	PACG	44.31 ± 1.89	44.26 ± 1.82	0.05 ± 0.16	−0.12, 0.02	0.134
	Control	45.04 ± 1.61	44.99 ± 2.62	−0.05 ± 0.10	−0.11, 0.01	0.082

WTW (mm)	PACG	11.45 ± 0.31	11.43 ± 0.25	−0.01 ± 0.14	−0.08, 0.05	0.705
	Control	11.34 ± 0.33	11.35 ± 0.30	0.01 ± 0.07	−0.03, 0.05	0.533

PD (mm)	PACG	3.45 ± 0.76	1.99 ± 0.46	−1.47 ± 0.46	−1.68, −1.25	<0.001
	Control	4.02 ± 0.53	1.70 ± 0.22	−2.32 ± 0.49	−2.59, −2.05	<0.001

ACD, anterior chamber depth; AL, axial length; CCT, central corneal thickness; D, diopter; LP, lens position; LT, lens thickness; PACG, primary angle-closure glaucoma; PD, pupil diameter; SD, standard deviation.

**Table 3:** Influence of pilocarpine eyedrops on IOL power calculated using five formulas in PACG patients and normal subjects.

Formulas	Group	Baseline	After eyedrops	Mean difference ± SD	95% CI for mean difference	*p* values
BU II	PACG	22.56 ± 2.03	22.56 ± 2.05	0.00 ± 0.20	−0.08, 0.09	0.940
	Control	22.64 ± 1.49	22.63 ± 1.50	−0.01 ± 0.17	−0.10, 0.09	0.833

Haigis	PACG	22.72 ± 2.14	22.74 ± 2.13	0.01 ± 0.21	−0.08, 0.11	0.745
	Control	22.69 ± 1.42	22.71 ± 1.44	0.02 ± 0.16	−0.08, 0.11	0.724

Hoffer Q	PACG	23.07 ± 2.26	23.10 ± 2.28	0.03 ± 0.20	−0.06, 0.12	0.474
	Control	22.89 ± 1.57	22.92 ± 1.59	0.03 ± 0.16	−0.06, 0.12	0.427

Holladay I	PACG	23.00 ± 2.10	23.02 ± 2.14	0.02 ± 0.22	−0.08, 0.12	0.670
	Control	22.85 ± 1.47	22.88 ± 1.49	0.03 ± 0.15	−0.05, 0.15	0.439

SRK/T	PACG	22.91 ± 2.03	22.95 ± 2.06	0.04 ± 0.19	−0.04, 0.12	0.346
	Control	22.88 ± 1.38	22.91 ± 1.39	0.02 ± 0.13	−0.05, 0.09	0.473

BU II: Barrett Universal II; IOL: intraocular lens; PACG: primary angle-closure glaucoma; SD: standard deviation.

**Table 4:** Coincidence of IOL power calculated using the five formulas in PACG patients and normal subjects before and after pilocarpine eyedrops instillation.

Coincidence	Group	Number of eyes				
		BU II	Haigis	Hoffer Q	Holladay I	SRK/T
Yes	PACG	19 (86%)	17 (77%)	18 (82%)	18 (82%)	19 (86%)
	Control	14 (93%)	12 (80%)	12 (80%)	13 (87%)	14 (93%)

No	PACG	3 (14%)	5 (23%)	4 (18%)	4 (18%)	3 (14%)
	Control	1 (7%)	3 (20%)	3 (20%)	2 (13%)	1 (7%)

BU II: Barrett Universal II; IOL: intraocular lens; PACG: primary angle-closure glaucoma.

## Data Availability

All the datasets supporting the discoveries are contained in this current manuscript and tables. The relevant raw data will be freely available from Xiamen University Affiliated Xiamen Eye Center by request.
